# Stress and autonomic nerve dysfunction monitoring in perioperative gastric cancer patients using a smart device

**DOI:** 10.1111/anec.12903

**Published:** 2021-10-20

**Authors:** Wei Cheng, Jiang Liu, Mengwei Zhi, Danli Shen, Mingyue Shao, Cheng Zhang, Gang Wang, Zhiwei Jiang

**Affiliations:** ^1^ Department of General Surgery Jiangsu Province Hospital of Chinese Medicine Affiliated Hospital of Nanjing University of Chinese Medicine Nanjing China; ^2^ School of Nursing Nanjing University of Chinese Medicine Nanjing China

**Keywords:** autonomic nerve dysfunction, gastric cancer, heart rate variability, inflammation biomarkers, plasma cortisol, stress

## Abstract

**Background:**

Heart rate variability (HRV), a sensitive marker of stress and autonomic nervous disorders, was significantly decreased in cardiovascular disease, inflammation, and surgical injury. However, the effect of radical gastrectomy on HRV parameters needs to be further investigated.

**Methods:**

A prospective, observational study including 45 consecutive enrolled patients undergoing radical gastrectomy in our enhanced recovery after surgery (ERAS) programs was conducted. Frequency‐ and time‐domain parameters of HRV from 1 day prior to operation to 4 days postoperatively were continuously measured. Meanwhile, plasma cortisol and inflammatory markers were recorded and correlated to HRV parameters.

**Results:**

Heart rate variability showed a solidly circadian rhythm. Anesthesia severely disturbed HRV parameters, resulting in a reduction of most of the HRV parameters. Frequency‐domain parameter (including VLF) and time‐domain parameters (including the SDNN, SDANN, and triangular index) of HRV demonstrated a significant reduction compared to preoperative values on the postoperative day 1 (Pod1), and these HRV parameters could return to baseline on Pod2 or Pod3, indicating surgical stress and autonomic nerve dysfunction existed in the early postoperative period. Inflammatory biomarkers were significantly elevated on Pod1 and Pod3. Plasma cortisol decreased significantly on Pod1 and Pod3. Both inflammatory biomarkers and plasma cortisol had no significant correlation with HRV parameters.

**Conclusions:**

Compared with plasma cortisol and inflammation biomarkers, HRV is more sensitive to detect surgical stress and autonomic nervous dysfunction induced by radical gastrectomy in patients with gastric cancer.

## 1 INTRODUCTION

Cancer is one of the major global threats to public health. Gastric cancer is the fourth leading cause of cancer‐related death worldwide (Latest Global Cancer Data). China is one of the countries with high incidence of gastric cancer (Latest Global Cancer Data; Wang et al., 2019). Surgery is the mainstay of treatment for many patients with gastric cancer. However, surgical intervention can also cause stress response and autonomic nerve dysregulation (Haase et al., 2012; Manou‐Stathopoulou et al., 2019). Even uncomplicated abdominal surgery could trigger dysfunction of the autonomic system, characterized by a relative decrease in parasympathetic tone (Colombo et al., 2017; Haase et al., 2012). Our previous studies demonstrated that patients undergoing gastrectomy in institutions applying enhanced recovery after surgery (ERAS) have an early convalescence (Wang et al., 2016; Zhao, Hu, Jiang, Wang, et al., 2018; Zhao et al., 2018), but the perioperative stress response and autonomic nerve dysregulation of these gastric cancer patients remain to be further studied.

Currently, a variety of methods can be used to evaluate stress, including the self‐administered questionnaire (SAQ) (He et al., 2018) and biochemical methods including the measurement of stress‐related substances such as plasma concentrations of cortisol (Manou‐Stathopoulou et al., 2019). SAQ is most frequently used, but it requires subjects to maintain their reflective capability. Therefore, SAQ is not suitable for assessing perioperative stress level. For stress‐related biochemical substances, there are significant differences in levels between individuals and throughout the circadian cycle. Therefore, it is difficult to reliably evaluate the perioperative real‐time pressure (Kwon et al., 2020; Zatti et al., 2019).

Heart rate variability (HRV) measurements, which were put forward by Hon and Lee for the first time in 1965 (Lee & Hon, 1965) are non‐invasive and standardized method to assess the stress response and autonomic nervous function (Ardissino et al., 2019; Charlier et al., 2020; Mulkey et al., 2020). Heart rates fluctuate continuously even at rest and are determined by the discharge cycle of the sinoatrial node. Sinoatrial node discharge is regulated by intracellular levels of potassium (K^+^) and calcium (Ca^2+^), both of which are regulated by autonomic nerves. The autonomic nerve activity can be evaluated by frequency and time‐domain HRV analysis of ECG data (Ptaszynski et al., 2013). In recent decades, HRV has aroused extensive clinical interest, mainly in the field of cardiovascular disease (Dias et al., 2019; Kališnik et al., 2019). Studies have shown that lower HRV was strongly associated with worse prognosis and higher risk of cardiac death (Goldenberg et al., 2019; Manresa‐Rocamora et al., 2021; Pinheiro et al., 2015). HRV has also become an effective prognostic tool in intensive care medicine (Joshi et al., 2020). However, there are no studies to evaluate gastrectomy‐induced stress and autonomic nerve dysfunction in gastric cancer patients by HRV. This study aimed to explore the circadian rhythm and fluctuation of HRV through a non‐invasive smart device that can continuously monitor HRV, further clarify the perioperative fluctuation of HRV, and evaluate the surgery‐induced stress and autonomic nerve dysfunction in gastric cancer patients.

## 2 METHODS

### 2.1 Patients

An observational study was conducted by a gastric surgery team of a first‐class hospital. Patients were admitted to the hospital consecutively from July 1, 2019, to October 31, 2020. Written informed consent was obtained from all patients prior to enrollment.

Inclusion criteria: (1) age: 50–75; (2) who was diagnosed as gastric cancer by histopathology and received 3D laparoscopic or Da Vinci Xi assisted radical gastrectomy. Exclusion criteria: (1) ASA IV~V level; (2) patients with arrhythmia, diabetes, or severe postoperative complications, including postoperative bleeding, anastomotic fistula.

As reported in our previous literature (Yun et al., 2018; Zhao, Hu, Jiang, Wang, et al., 2018), patients were managed perioperatively in the ERAS programs applied for gastrectomy in our department, including preoperative and postoperative nutritional support, multi‐mode health education, no intestinal preparation for non‐constipated patients, shortened preoperative fasting time and oral carbohydrate administration, patients temperature maintenance, limited fluid resuscitation, postoperative pain assessment and multimodal analgesia, early mobilization, and early oral feeding.

### 2.2 Data collection

**FIGURE 1:**
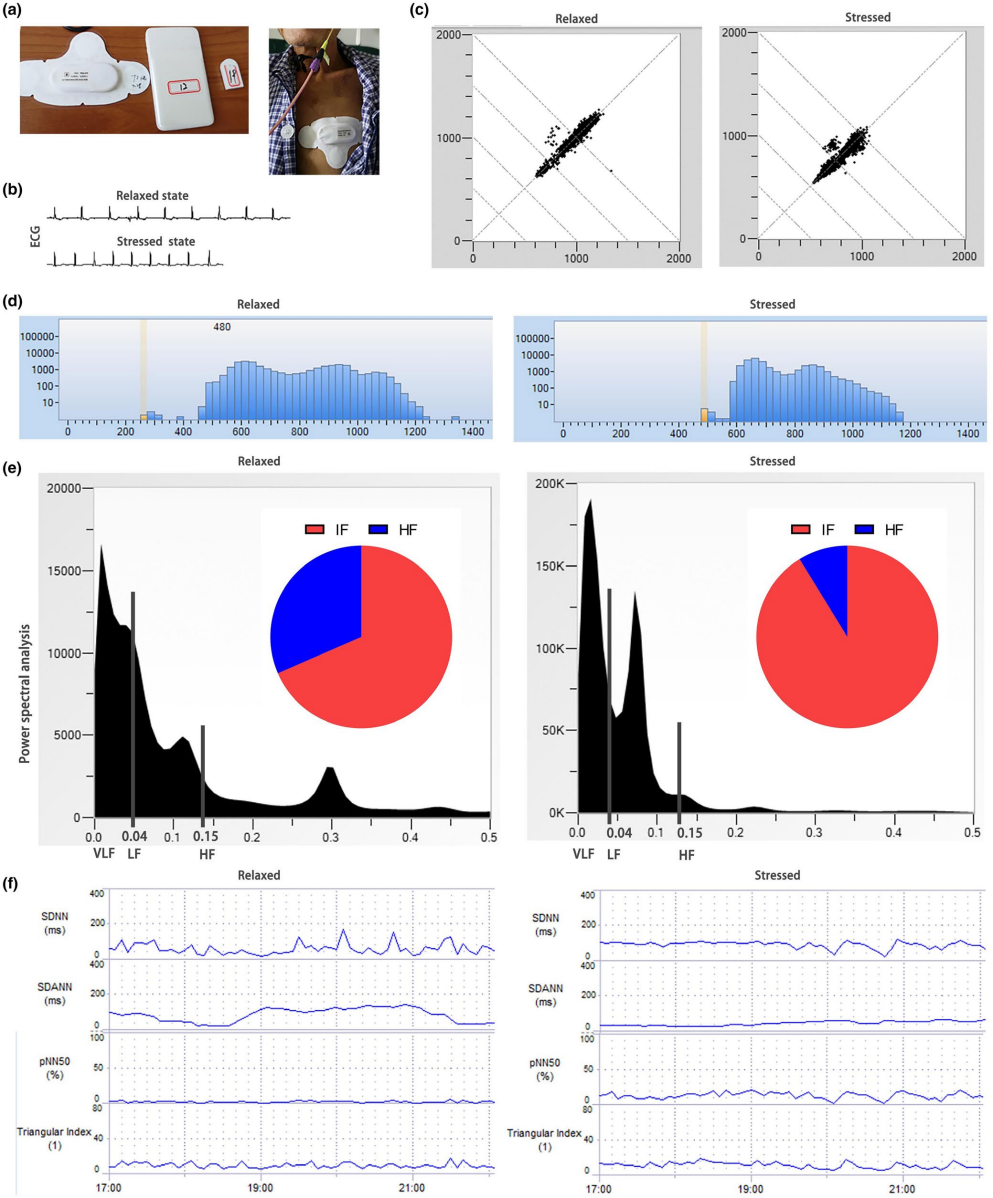
Heart rate variability (HRV) in a relaxed state and under severe mental stressed state. (a) A smart monitoring device for the collection of HRV data; (b) ECG manifestations in relaxed and stressed state; (c) Poincaré scatter diagram in a relaxed and stressed state; (d) Histogram indicates the difference between relaxed and stressed state; (e) Power spectral density analysis of frequency‐domain analysis indicates the difference between relaxed and stressed state, including power of VLF spectrum (<0.04 Hz in frequency band), power of LF spectrum (0.04–0.15 Hz in frequency band), power of HF spectrum (0.15–0.40 Hz in frequency band), and the ratio of LF to HF (LF/HF); (f) Trend chart of time‐domain parameters indicate the difference between relaxed and stressed state. HF, high‐frequency power; LF, low‐frequency power

A smart device (Transcendent THoughts On Health, THOTH, http://www.thoth‐health.com/) was used for HRV monitoring. After the patients were admitted to the hospital, the researchers cleaned the skin of the precardiac area with wet gauze, attached the registered smart device to the skin in the shape of T (Figure 1a). And then the full‐course heart rate and HRV were monitored from 1‐day pre‐operation (Pre) to 4 days post‐operation (Pod4). After the data collection, the Holter System software was used for statistical analysis (Figure 1b‐f).

In addition to HRV, collected variables included gender, age, body mass index, duration of surgery, bleeding volume, white blood cell count, neutrophil percentage, CRP, PCT, plasma cortisol, surgical complications,and et al.

### 2.3 HRV analysis

Heart rate variability measurement methods include Poincaré plot (Figure 1c), R–R interval histogram (Figure 1d), frequency‐domain analysis, and time‐domain analysis (Figure 1e‐f), among which time‐domain and frequency‐domain analysis methods are most widely used in clinical practice. Time‐domain analysis method is to collect the values of successive sinus R–R interval and conduct statistical analysis according to the time or sequence of cardiac beats. Analyzed time‐domain parameters included the standard deviation of all NN intervals (SDNN), standard deviation of all the averages of NN intervals (SDANN) in all 5‐min segments of the entire recording, triangular index, and NN50 count (number of pairs of adjacent NN intervals differing by >50 ms in the entire recording) divided by the total number of all NN intervals (pNN50). Analyzed frequency‐domain analysis includes very‐low‐frequency power (VLF), low‐frequency power (LF), high‐frequency power (HF), and the ratio of low‐frequency power to high‐frequency power (LF/HF). In this study, one datum is obtained every hour in time‐domain analysis, and each data is the statistical result of all the obtained data in this hour. In frequency‐domain analysis, one data is obtained in one hour, which is a statistical result of five minutes of monitored data in that hour.

Even in the resting state, human's HRV fluctuates regularly and varies with circadian rhythms. Therefore, we first analyzed the preoperative circadian rhythms of HRV according to the data obtained from the continuous monitoring.

**FIGURE 2:**
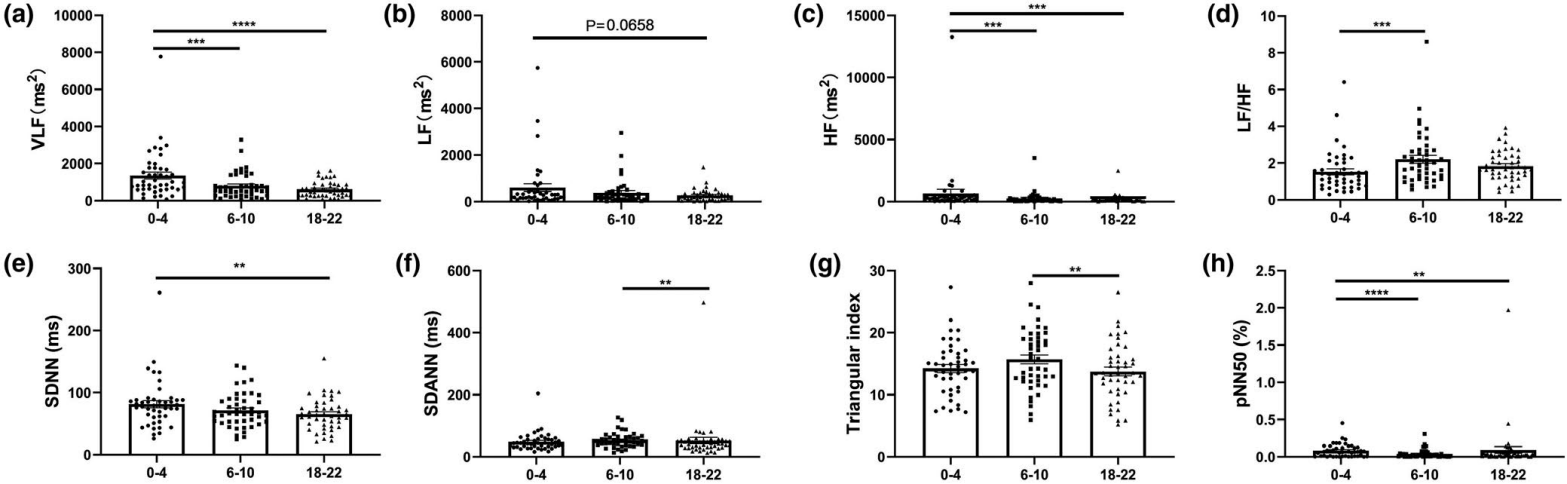
Preoperative circadian rhythm of heart rate variability. According to sleep–wake cycle and activity level difference, one day is divided into three periods (0–4, 6–10, 18–22). (a‐d) circadian rhythm of frequency‐domain analysis parameters of heart rate variability (HRV), including VLF, LF, HF and the ratio of low‐frequency power to high‐frequency power (LF/HF); (e‐h) circadian rhythm of time‐domain analysis parameters of HRV, including SDNN, SDANN, triangular index, and pNN50, *N* = 45. **p < .01, ***p < .001, ****p < .0001. The data were represented as scatter plot and mean ± standard error. HF, high‐frequency power; LF, low‐frequency power ; VLF, very‐low‐frequency power; SDNN, standard deviation of all NN intervals; SDANN, standard deviation of all the averages of NN intervals

Then, according to the result of preoperative circadian rhythm analysis, we found that HRV data of 0 to 4 o'clock (0, 1, 2, 3, 4) in the morning showed good consistency, HRV data of 6 to 10 o'clock (6, 7, 8, 9, 10) showed good consistency, and HRV data of 18 to 22 o'clock (18, 19, 20, 21, 22) also showed good consistency. In addition, there are statistical differences in some parameters of both frequency‐domain and time‐domain HRV among 0–4, 6–10, and 18–22 (Figure 2). Therefore, based on these results, we conducted statistical analysis of perioperative time‐division HRV data (0–4, 6–10, and 18–22) on and throughout day HRV data, and comprehensively evaluated the changes of perioperative HRV in patients undergoing gastrectomy.

### 2.4 Statistical analysis

Statistical analysis was performed using GraphPad Prism software (version 8). All HRV data are shown as the mean ± standard error of the mean (mean ± SEM). Normality of data distribution was tested using the Shapiro‐Wilk test. The significance of differences of repeated measures of HRV parameters was evaluated using the Friedman test and Dunn's multiple comparisons test as the post hoc test. Spearman's rank correlation coefficient was also used to evaluate the correlation between perioperative HRV parameters and white blood cell count, Neutrophil percentage (%), CRP, PCT, or plasma cortisol. Significance was defined as *p ≤ .05*.

## 3 RESULTS

### 3.1 Patient characteristics

In this study, 45 patients who underwent radical gastrectomy for gastric cancer were enrolled consecutively, with a median age of 61 (50–75) years, including 4 females and 41 males. The body mass index was 22.2 ± 2.8 kg/m^2^ (mean ± SD). All patients were treated by the same team. The operations were assisted by 3D laparoscopic (*n* = 21) and Da Vinci Xi robot (*n* = 24), including 13 proximal gastrectomy, 20 distal gastrectomy, and 12 total gastrectomy. The operation time was 231.0±50.5 (mean ± SD) minutes and the mean bleeding volume was 60.2 ± 4.8 ml. The clinical stage is shown in Table 1. In addition, there were two surgical complications, including one delayed gastric emptying and one surgical site infection. The detailed demographics are shown in Table 1.

**TABLE 1:** Patients' demographics

Parameter	*N* = 45	%
Median age (range)	61 (50–75)	
Male	41	91.1%
Mean body mass index (±SD)	22.2 ± 2.8 kg/m^2^	
Assistive devices		
3D‐laparoscopic	21	46.7%
Da Vinci Xi	24	53.3%
Surgical method		
Proximal gastrectomy	13	28.9%
Distal gastrectomy	20	44.4%
Total gastrectomy	12	26.7%
Mean operation time (±SD)	231.0 ± 50.5 mins	
Mean bleeding volume (±SD)	60.2 ± 4.8 ml	
Clinical stage		
I	14	31.1%
II	12	26.7%
III	19	42.2%
Complications		
Delayed gastric emptying	1	2.2%
Surgical site infection	1	2.2%

### 3.2 Preoperative circadian rhythm of heart rate variability

The frequency‐domain analysis found that VLF, LF, and HF showed consistent preoperative circadian rhythm. Compared with the time periods of 6–10 or 18–22, VLF and HF were statistic higher at 0–4 (Figure 2a,c). Although there was no statistical difference, LF was actually higher at 0–4 than at the other two periods (Figure 2b). VLF, LF, and HF at 18–22 had no statistical difference when compared with that at 6–10 (Figure 2a‐c). By contrast, the LF/HF at 6–10 was significantly higher than that at 0–4 (Figure 2d).

According to the time‐domain analysis, SDNN of 0–4 was statistically higher than that of 18–22 (Figure 2e). SDANN and triangular index showed the same circadian rhythm, and these two parameters were highest at 6–10, which were significantly higher than that at 18–22 (Figure 2f‐g). The pNN50 was the highest at 0–4, which was significantly higher than those at 6–10 and 18–22 (Figure 2h).

### 3.3 Perioperative HRV analysis

**FIGURE 3:**
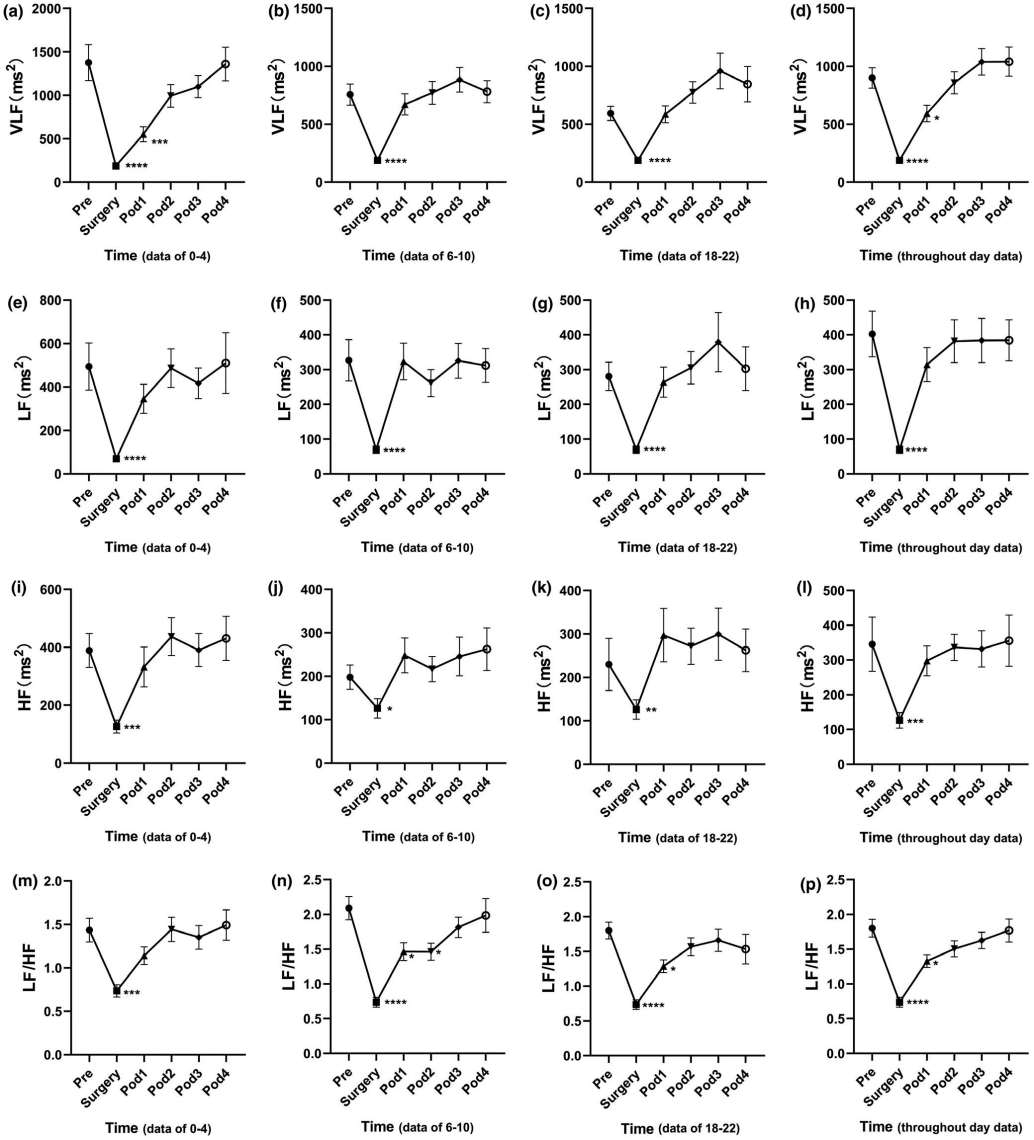
Fluctuation of frequency‐domain heart rate variability (HRV) analysis parameters in perioperative period in patients undergoing laparoscopic radical gastrectomy. (a\e\i\m) are data of 0–4, (b\f\j\n) are data of 6–10, (c\g\k\o) are data of 18–22, (d\h\l\p) are throughout day data. *N* = 45. *p < .05, **p < .01, ***p < .001, ****p < .0001. The data were represented as mean ± standard error

**FIGURE 4:**
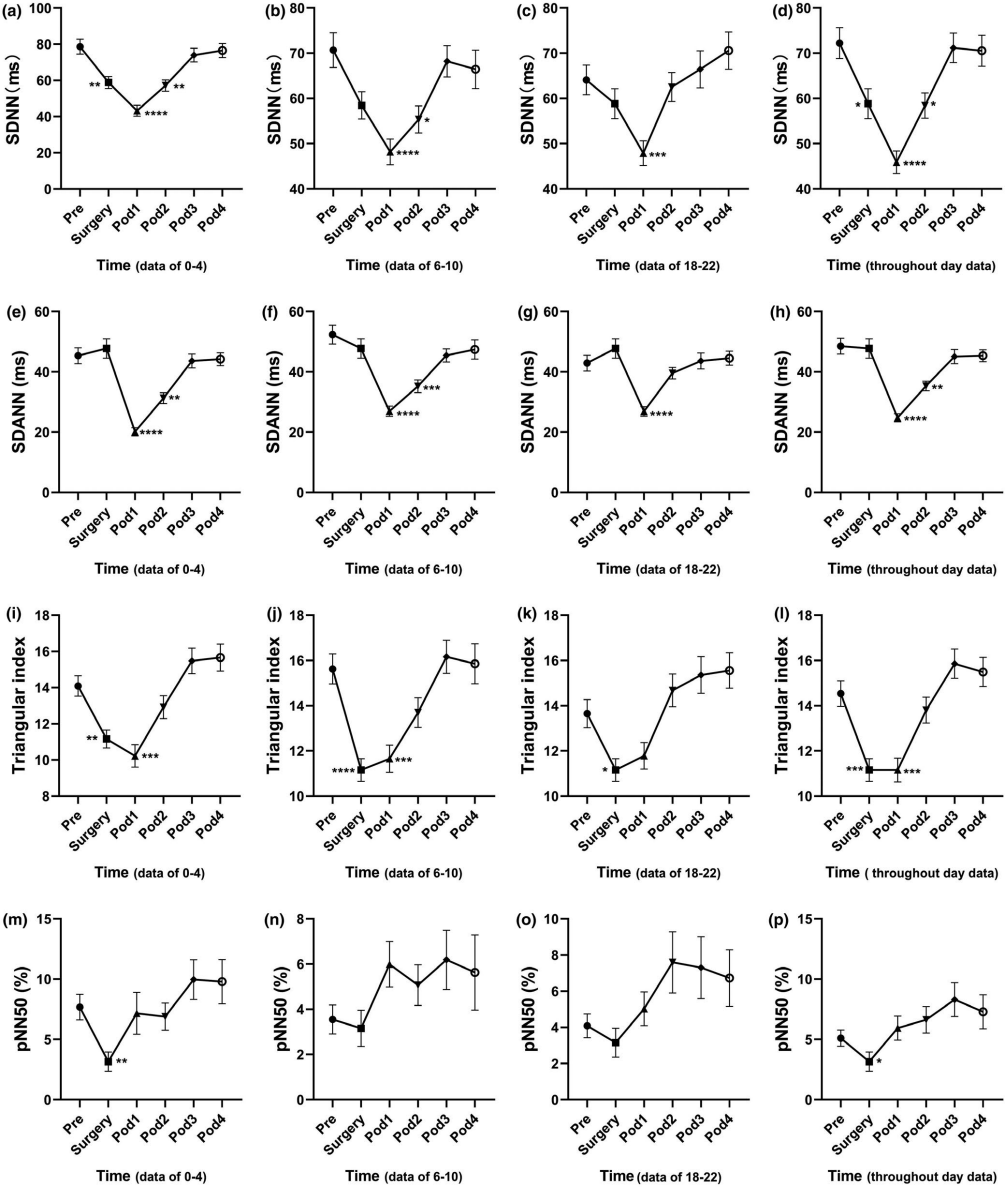
Fluctuation of time‐domain heart rate variability (HRV) analysis parameters in perioperative period in patients undergoing laparoscopic radical gastrectomy. (a\e\i\m) are data of 0–4, (b\f\j\n) are data of 6–10, (c\g\k\o) are data of 18–22, (d\h\l\p) are throughout day data. *N* = 45. *p < .05, **p < .01, ***p < .001, ****p < .0001. The data were represented as mean ± standard error

For all the perioperative parameters of HRV, the throughout day data and the time‐division data (including data of 0–4, or 6–10 or 18–22) showed similar trends and could recover to preoperative levels at post‐operation day3 (Pod3), although a certain degree of difference existed (Figures 3 and 4). All the frequency‐domain HRV parameters (including VLF, LF, HF, and LF/HF) decreased significantly during the operation, demonstrated by both the time‐division data and throughout day data (Figure 3). The data of 0–4 and throughout day data showed that the VLF decreased significantly at Pod1 compared with Pre and returned to normal at Pod2 (Figure 3a,d). For LF/HF, the data of 18–22 and throughout day data and showed significantly decreased at Pod1 compared with Pre and returned to normal at Pod2 (Figure 3o,p). In addition, the LF/HF data of 6–10 showed a significant reduction at Pod1 and Pod2 and could return to the preoperative level at Pod3 (Figure 3n).

Standard deviation of all NN intervals, Triangular index, and pNN50 were significantly decreased during anesthesia in the surgery, indicating that anesthesia might significantly affect the time‐domain parameters (Figure 4a, d, i‐l, m, p). Interestingly, there was no significant difference of SDANN in surgery demonstrated by both the time‐division data and throughout day data, suggesting that anesthesia did not affect SDANN in patients (Figure 4e‐h). SDNN and SDANN were remarkably decreased at Pod1 and Pod2, and these two parameters returned to normal at Pod3 (Figure 4a, b, d, e, f, and h). Triangular Index was decreased at Pod1 and could return to the preoperative levels at Pod2, indicating a faster recovery. Compared with Pre, pNN50 showed some fluctuations at Pod1‐Pod4, but there was no statistically significant difference (Figure 4m‐p).

### 3.4 Perioperative inflammatory markers and plasma cortisol

Compared with pre‐operation, all inflammatory markers (including WBC, Neutrophil percentage, CRP, and PCT) increased significantly at Pod1 and Pod3. WBC and Neutrophil percentages decreased significantly at Pod3 as compared with Pod1, indicating a markedly attenuated inflammatory reaction (Table 2). However, although CRP and PCT were lower at Pod3 than Pod1, they were not significantly decreased, suggesting a more sensitive and persistent response to the surgical inflammatory injury (Table 2). We collected blood from 5 patients to measure their plasma cortisol levels and found that the plasma cortisol decreased significantly at both Pod1 and Pod3. Subsequently, plasma cortisol increased significantly at Pod3 when compared with Pod1 (Table 2).

**TABLE 2:** Statistics of inflammation‐related parameters and plasma cortisol

Parameters	Pre	Pod1	Pod3
WBC (10^9^, *N* = 38)	4.95 ± 1.70	11.67 ± 4.76****	7.58 ± 2.75****^,△△△△^
Neutrophil percentage (%, *N* = 38)	57.77 ± 10.36	86.23 ± 4.70****	72.07 ± 9.57****^,△△△△^
CRP (mg/dl, *N* = 21)	2.68 ± 1.46	34.58 ± 25.41****	26.45 ± 20.76****
PCT (ng/ml, *N* = 30)	0.051 ± 0.030	0.599 ± 0.599****	0.564 ± 1.563**
Cortisol (μg/dl, *N* = 5)	11.88 ± 5.97	5.50 ± 2.52***	8.5 ± 3.92*^,△^

Note

*p < .05 vs. Pre, ^△^ *p* < .05 vs. Pod1.

Abbreviations: CRP, C‐reactive protein; PCT, procalcitonin; WBC, white blood cell.

Further, we analyzed the correlation between HRV and inflammatory markers or plasma cortisol. The changes of HRV parameters were not correlated with the elevation of inflammatory biomarkers (including white blood cell count, neutrophil percentage, CRP, and PCT) or plasma cortisol at both POD1 and POD3 (*p* > .05).

## 4 DISCUSSION

### 4.1 Circadian rhythm of heart rate variability

Heart rate variability is affected by the sleep–wake cycle and activity (Hattori & Asamoto, 2020; Ho et al., 2020; Ptaszynski et al., 2013; Sűdy et al., 2019; Urbanik et al., 2019). Therefore, to explore the circadian rhythm of HRV, the preoperative HRV data obtained from continuous monitoring were analyzed according to different time‐division (including deep sleep period at 0–4 early in the morning, the daytime active stage at 6–10, and the evening recreational period at 18–22). Based on this methodology, more accurate and detailed perioperative data of HRV could be obtained, and then comprehensively understand the postoperative autonomic nerve activity and surgical stress of patients. A study by H. Israelilovic suggests that 24‐h continuous monitoring of HRV by smart device is non‐invasive, reliable, feasible, and not expensive, and maybe important for early identification of sepsis stress (Israeli‐Mendlovic et al., 2020). Our results demonstrated that VLF, LF, and HF were higher at 0–4, suggesting that autonomic nervous function was more active in deep sleep state. LF/HF was higher in the daytime active stage at 6–10, which indicated that the sympathetic nervous system activity was relatively enhanced in the daytime active stage. In addition, the time‐domain parameter pNN50 showed that the patients had significantly higher vagus nerve activity at 0–4, indicating that the parasympathetic nerve was more active at deep sleep state. SDNN reflects total heart rate variability, and the higher SDNN indicates better health and physiological resilience, which are negatively correlated with cardiac risk (Lopresti, 2020; Shaffer & Ginsberg, 2017). Our study showed that SDNN peaked at 0–4, suggesting that sleep and rest might be important for maintaining physical health. SDANN and triangular index showed similar circadian rhythms and peaked at 6–10. Studies have shown a high positive correlation between SDNN and SDANN (Lopresti, 2020), but our study solidly proved that the circadian rhythms of SDNN and SDANN were not consistent and they peaked at different time period, rising a problem that probably deserves more attention in further study. These results strongly demonstrated that HRV had a circadian rhythm. Therefore, we need to pay attention to variables control when studying HRV, including controlling the monitoring time point of HRV and controlling the consistency of activity intensity. If HRV parameters are obtained in the natural state, HRV should better be 24‐hour continuously monitored, and analyzed according to different time division.

### 4.2 Perioperative HRV of gastric cancer patients

According to the analyzed parameters of HRV, anesthesia seriously disturbed both the time‐division data and throughout day data of HRV parameters, manifested as significant reduction of VLF, LF, HF, SDNN, triangular index, and pNN50. But interestingly, SDANN was the only HRV parameter that was not significantly affected by anesthesia, indicating that SDANN was quite special, and further demonstrated that SDANN and SDNN were not completely positively correlated. Studies by Mathieu Jeanne, Maddalena Ardissino, and Thomas Ledowski have also demonstrated that anesthesia could result in significant reductions of multiple HRV parameters, which is consistent with our findings (Ardissino et al., 2019; Jeanne et al., 2009; Ledowski et al., 2005). Meanwhile, it was clear that the HRV at POD1 and POD2 is significantly reduced, presented as significant reduction of SDNN and SDANN, indicating patients suffering of a significant postoperative stress at POD1 and POD2, and implicating suppression of autonomic nervous system activity (Garg et al., 2020; Hattori & Asamoto, 2020; Tracy et al., 2016). Moreover, the reduction of LF/HF, which was used to evaluate the balance of sympathetic and parasympathetic nervous system (Lopresti, 2020), further suggesting that the sympathetic/parasympathetic imbalance occurred during the perioperative period. HF is a well‐known HRV parameter that can reflect the parasympathetic and vagal activity (Lopresti, 2020). By continuous monitoring, we did not find any significant difference between the postoperative HF and the preoperative HF, indicating that the postoperative vagal activity of the patients was not significantly inhibited. pNN50, which is highly positive correlated with HF, also reflects parasympathetic and vagal activity. We found no significant difference of pNN50 between the postoperative and preoperative data, which further illustrated unchanged vagal activity of gastric cancer patients undergoing surgical treatment in our ERAS program. This might benefit from the multimodal analgesia in our ERAS program (Zhao, Hu, Jiang, Wang, et al., 2018; Zhao et al., 2018). Most of our patients could perform off‐bed activities independently on the first day after surgery, and even not feel a thing. At Pod3, all parameters of HRV returned to the preoperative level, indicating that the postoperative stress state of patients had been gradually eliminated. This is consistent with the previous research results. Haase and colleagues discussed the changes of HRV after colorectal cancer resection under perioperative “fast‐track” management. The research results showed that all parameters of HRV decreased significantly on the first day after the surgery, and all recovered to the preoperative level on the second or third day after the surgery (Haase et al., 2012).

### 4.3 Perioperative inflammatory markers and plasma cortisol

In addition, perioperative inflammatory markers were also detected. WBC and Neutrophil percentage increased significantly on the first day after surgery and decreased significantly on the third day. However, CRP and PCT were significantly increased on the first day after surgery, but not significantly decreased on the third postoperative day. Further, we analyzed the correlation between these inflammatory markers and HRV parameters. However, this study showed no correlation between any postoperative parameters of HRV and any inflammatory markers, suggesting that the inflammatory biomarkers could not reflect the postoperative stress, assumed that HRV was the best means to monitor surgical stress. And these results were consistent with the findings in patients with colorectal resections by Haase et al., (2012). Moreover, the plasma cortisol was not correlated with any HRV parameters. Cortisol is a marker of physiological stress, and large surgical stress may trigger a significant increase in plasma cortisol (Dimopoulou et al., 2008; Kapritsou et al., 2020). However, a growing number of studies in recent years have suggested that with the advancement of minimally invasive surgery, anesthesia and analgesia techniques, surgery may not lead to a significant increase in postoperative cortisol (Khoo et al., 2017; Prete & Yan, 2018). Similarly, laparoscopic surgery may not lead to a significant increase in postoperative cortisol. As in this study, there was a dramatically decrease in postoperative cortisol. This may be attributed to our minimally invasive laparoscopic surgery (including 3D laparoscopic and Da Vinci robotic surgery), multimodal analgesia and early removal of drainage, which minimize postoperative pain stress in the ERAS program (Schreiber et al., 2019; Zhao, Hu, Jiang, Wang, et al., 2018; Zhao et al., 2018c). Consistent with this study, Li Ren also confirmed that ERAS protocol could significantly reduce the postoperative serum cortisol level, so that the postoperative serum cortisol did not increase significantly compared with that before surgery (Ren et al., 2012). But in reality, surgical stress does exist. This study found that the plasma cortisol was not sensitive to detect the patient's postoperative stress, but the time‐domain parameters of HRV (including SDNN, SDANN, and triangular index) were extraordinarily sensitive to confirm the patient's postoperative stress. These results suggest that HRV is a more accurate indicator of postoperative stress in surgical patients than cortisol. On the other hand, our team has started to routinely monitor the perioperative HRV and plasma cortisol of all HRV parameters in patients with gastric cancer and colorectal cancer, in ERAS program or non‐ERAS program. Therefore, a larger sample size study of perioperative stress is on the way.

### 4.4 Advantages and limitations

The advantage of this study is the 24‐h continuous whole‐course monitoring of HRV, which is convenient to observe the change of HRV throughout the perioperative period and capture valuable difference data. Moreover, the use of smart wearable medical devices and wireless communication technology for data collection can reduce the inconvenience to patients' movement and greatly improve patients' compliance and the accessibility to data.

Limitations of this study are as follows: the age range of patients we included was narrow in consideration of the influence of patients' age on HRV, which led to a relatively small number of participants in the study. In future, our research team will carry out relevant studies on surgical stress of gastric cancer, colorectal cancer, and gallbladder stones in our ERAS programs, so as to monitor and reduce the postoperative stress of patients and promote their rapid recovery after surgery. Therefore, large sample and long‐term observation studies are already on the way to explore the effects of pain, fasting, and sleep disorders on perioperative HRV, so as to guide us to improve our ERAS program.

## 5 CONCLUSIONS

This study demonstrated that preoperative HRV of gastric cancer patients had circadian rhythm, and the obtained HRV data needed throughout day data and time‐division data analysis, so as to comprehensively and accurately obtain the changes of perioperative HRV and surgical stress of patients. Perioperative heart rate variability monitoring revealed that anesthesia would disturb the HRV, resulting in a reduction of most of the HRV parameters. HRV monitoring showed a decrease in HRV parameters in the early postoperative period, indicating the existence of postoperative stress. Doctors should try their best to reduce perioperative stress and enhance patients' recovery through minimally invasive surgery and multimodal analgesia et al. in the ERAS program. However, the plasma cortisol was significantly reduced after surgery, so this parameter was not sensitive to reflect postoperative stress. In comparison, HRV could objectively reflect the changes in autonomic nerve function and stress response during perioperative period of gastric cancer and might be used as a valuable tool to evaluate perioperative stress response and guide clinical practice in the context of precision medicine and artificial intelligence medicine.

## CONFLICT OF INTEREST

The authors report no conflicts of interest in this work.

## AUTHOR CONTRIBUTIONS

Zhiwei Jiang and Gang Wang: conceived and designed the study. Wei Cheng, Jiang Liu, Mengwei Zhi, and Danli Shen: conducted the experiments. Wei Cheng, Jiang Liu, Mingyue Shao, and Cheng Zhang: analyzed data. Wei Cheng and Jiang Liu: wrote the manuscript. All authors read, edited, and approved the manuscript.

## ETHICAL APPROVAL

Ethical approval for this study was granted by the ethics committee of Jiangsu Province Hospital of Chinese Medicine. It was also conducted according to the China Good Clinical Practice in Research.

## CONSENT TO PARTICIPATE

Written informed consent was obtained from all individual participants included in the study.

## CONSENT FOR PUBLICATION

Additional informed consent was obtained from all individual participants for whom identifying information is included in this article.

## Data Availability

The data that support the findings of this study are available from the corresponding author upon reasonable request.
